# Correction: ‘Sequential buckling mechanics of the ermine moth's aeroelastic tymbal: an origami-like creased shell analogue’ (2026) by Mendoza Nava *et al*.

**DOI:** 10.1098/rsif.2026.0946

**Published:** 2026-10-14

**Authors:** Hernaldo Mendoza Nava, Marc Holderied, Alberto Pirrera, Rainer Maria Johannes Groh

**Affiliations:** ^1^ Bristol Composites Institute, University of Bristol, Bristol, UK; ^2^ School of Biological Sciences, University of Bristol, Bristol, UK; ^3^ Department of Aerospace Engineering, University of Bristol, Bristol, UK

*J. R. Soc. Interface* **23**, 20250864 (Published online 1 February 2026). (https://doi.org/10.1098/rsif.2025.0864).

In the original version of the article, some funding information was accidentally omitted in the production process. The full funding statement is as follows.

## Funding

H.M.N. acknowledges financial support by the Science and Technology National Council (CONACYT - Mexico, CVU/Studentship No. 530777/472285). H.M.N. also acknowledges the support of the Engineering and Physical Sciences Research Council (EPSRC) through the Centre for Doctoral Training in Advanced Composites for Innovation and Science (grant no. EP/L0160208/1). A.P. acknowledges the support of EPSRC through an Early-Career Fellowship (grant no. EP/M013170/1). R.M.J.G. acknowledges the support of the Royal Academy of Engineering through the Research Fellowship scheme (grant no. RF/201718/17178).

